# Properties of Gaseous Deprotonated L-Cysteine S-Sulfate Anion [cysS-SO_3_]^−^: Intramolecular H-Bond Network, Electron Affinity, Chemically Active Site, and Vibrational Fingerprints

**DOI:** 10.3390/ijms24021682

**Published:** 2023-01-14

**Authors:** Qiaolin Wang, Zhengbo Qin, Gao-Lei Hou, Zheng Yang, Marat Valiev, Xue-Bin Wang, Xianfeng Zheng, Zhifeng Cui

**Affiliations:** 1 Anhui Province Key Laboratory of Optoelectric Materials Science and Technology, Anhui Normal University, Wuhu 241000, China; 2 Physical Sciences Division, Pacific Northwest National Laboratory, 902 Battelle Boulevard, MS K8-88, P.O. Box 999, Richland, WA 99352, USA; 3 Environmental Molecular Sciences Laboratory, Pacific Northwest National Laboratory, P.O. Box 999, Richland, WA 99352, USA

**Keywords:** hydrogen bond, photoelectron spectroscopy, vibrational fingerprint, biomolecule

## Abstract

L-cysteine S-sulfate, Cys-SSO_3_H, and their derivatives play essential roles in biological chemistry and pharmaceutical synthesis, yet their intrinsic molecular properties have not been studied to date. In this contribution, the deprotonated anion [cysS-SO_3_]^−^ was introduced in the gas phase by electrospray and characterized by size-selected, cryogenic, negative ion photoelectron spectroscopy. The electron affinity of the [cysS-SO_3_]^•^ radical was determined to be 4.95 ± 0.10 eV. In combination with theoretical calculations, it was found that the most stable structure of [cysS-SO_3_]^−^ (**S_1_**) is stabilized via three intramolecular hydrogen bonds (HBs); i.e., one O−H⋯⋯N between the –COOH and –NH_2_ groups, and two N−H⋯⋯O HBs between –NH_2_ and –SO_3_, in which the amino group serves as both HB acceptor and donor. In addition, a nearly iso-energetic conformer (**S_2_**) with the formation of an O−H⋯⋯N−H⋯⋯O−S chain-type binding motif competes with **S_1_** in the source. The most reactive site of the molecule susceptible for electrophilic attacks is the linkage S atom. Theoretically predicted infrared spectra indicate that O−H and N−H stretching modes are the fingerprint region (2800 to 3600 cm^−1^) to distinguish different isomers. The obtained information lays out a foundation to better understand the transformation and structure–reactivity correlation of Cys-SSO_3_H in biologic settings.

## 1. Introduction

L-cysteine S-sulfate and its derivatives are an important class of biochemical species via covalent attachment of a sulfate group (sulfation) to cysteine or cystine in post-translational modifications and play indispensable roles in metabolisms and pharmaceutical synthesis [1]. The general function of thiol S-sulfation is to affect the tissue hydration, cellular adhesive processes, (in)activation, and plasma clearance of small biomolecules [2]. It is also evidenced that both molybdenum co-factor deficiencies and isolated sulfite oxidase deficiencies stem from elevated urinary S-sulfocysteine levels [1]. A recent tandem mass spectrometry and dynamics simulations indicated that gaseous deprotonated L-cysteine S-sulfate anion [cysS-SO_3_]^−^ is a precursor for the generation of the cysteine sulfenic intermediate [cysSO]^−^ via low-energy collision-induced dissociation [3]. This pioneering, mass spectrometry-based observation has stimulated spectroscopic characterization of the electronic structure of [cysSO]^−^, proven to be a distonic radical anion [4]. Despite the importance of [cysS-SO_3_]^−^ in numerous biological processes, its intrinsic molecular properties have not yet been characterized. 

Gas-phase size-selective ion spectroscopy is a powerful method to investigate the geometries and electronic structures of a broad range of ionic species pertinent to electrolyte solutions, atmospheric chemistry, and biological processes [5,6,7]. It is also well documented that energy landscapes, structures, and electronic properties of those ionic species and clusters are critically dependent on the number and type of hydrogen bonds formed [8,9,10]. Herein, we report an integrated ion spectroscopic and theoretical study on the intrinsic molecular properties of [cysS-SO_3_]^−^. Our study illustrates the existence of a plethora of low-lying isomers for [cysS-SO_3_]^−^ driven by distinctly different intramolecular hydrogen bonds and identifies the most chemically active site residing on the linkage S atom.

## 2. Results and Discussion

**Figure 1:**
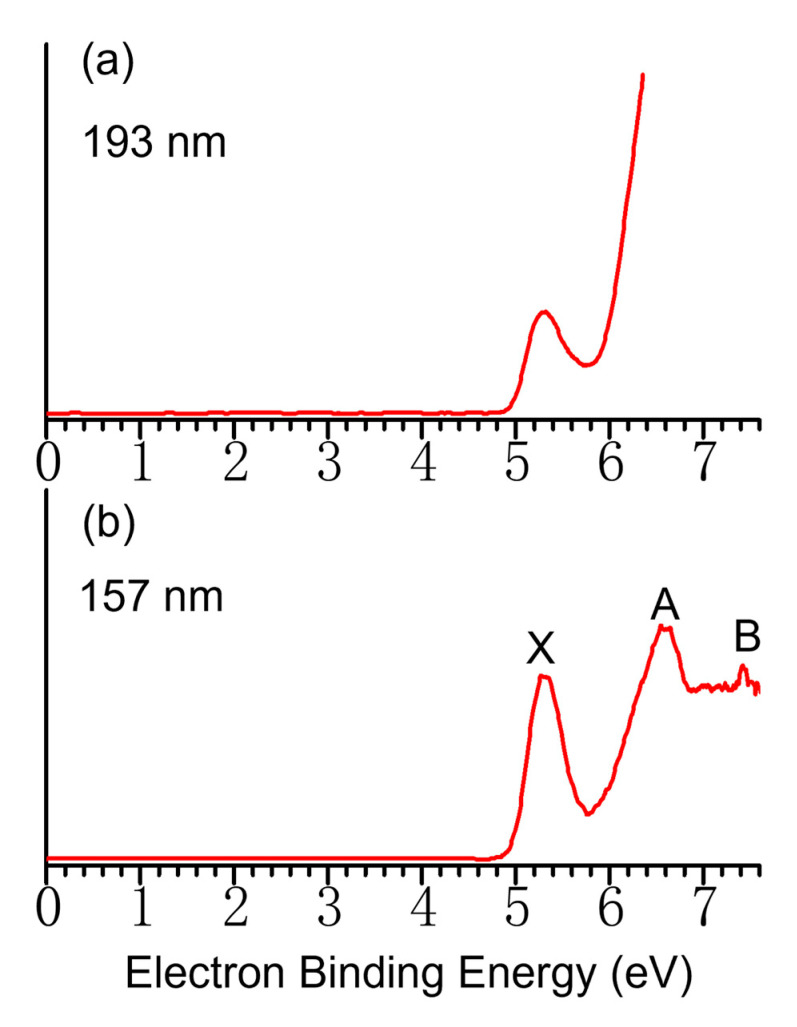
The 20 K NIPE spectra of the cysteine perthiosulfonate anion [cysS-SO_3_]^−^ at (**a**) 193 nm (6.424 eV) and (**b**) 157 nm (7.867 eV).

The 20 K photoelectron spectra of [cysS-SO_3_]^−^ taken with 193 nm and 157 nm laser are presented in Figure 1. Three spectral bands—X, A, and B—are observed at 157 nm in the EBE range of 5.0 to 7.6 eV, while only band X and the rising edge of band A are seen in the 193 nm spectrum. These spectral bands correspond to the transitions from the ground electronic state of the anion to the ground (X) and excited states (A, B) of the neutral. The experimental adiabatic detachment energy (ADE) was estimated from the spectral onset threshold to be 4.95 ± 0.10 eV, which is equivalent to the electron affinity (EA) of the [cysS-SO_3_]^•^ radical. The X band peak position of 5.30 ± 0.10 eV was assigned to the experimental vertical detachment energy (VDE) (see Table 1), corresponding to the photodetachment transition energy at which the Franck–Condon overlap between the anion and neutral species is largest. The VDE for band A was measured as 6.60 ± 0.10 eV. 

**Figure 2:**
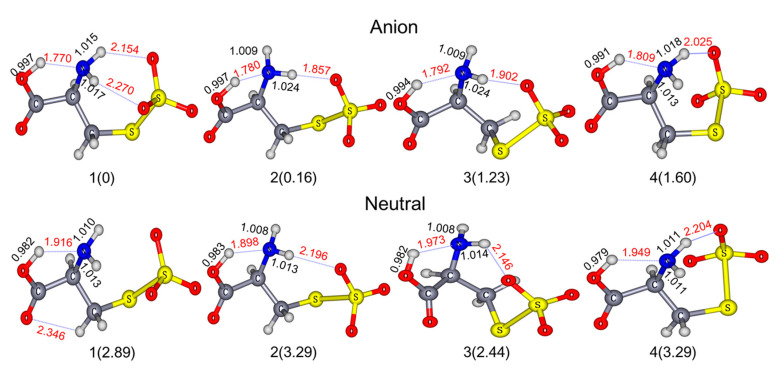
The top four lowest-lying energy conformers of cysteine perthiosulfonate anion [cysS-SO_3_]^−^ along with its corresponding neutral counterparts.

**Figure 3:**
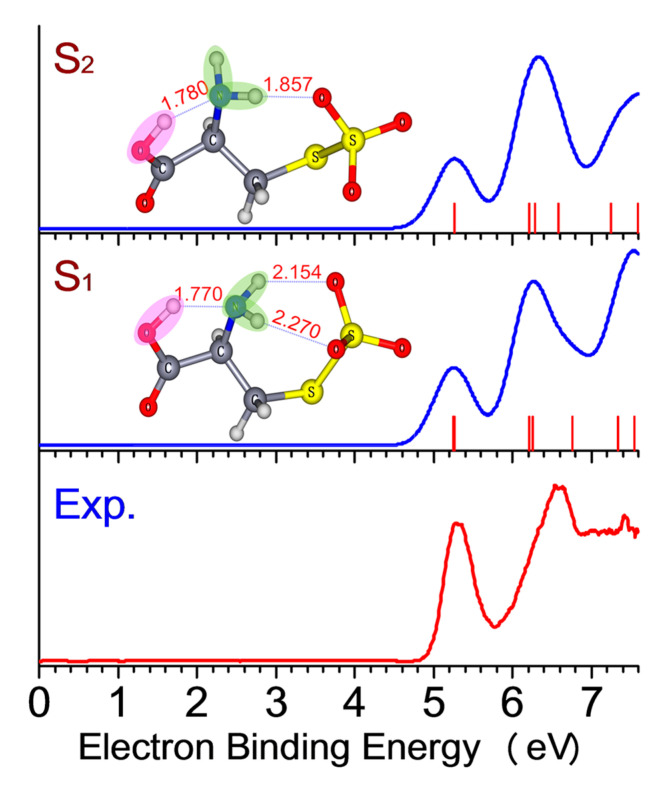
Comparison of the experimental spectrum (red curve) with simulated stick (thin red) and convoluted spectra (blue curves) of the most stable, nearly iso-energetic conformers of [cysS-SO_3_]^−^ (**S_1_** and **S_2_**). The simulations were based on the time-dependent (TD) CAM-B3LYP calculations using Gaussians with full widths at a half maximum of 350 meV and the first stick that represents the transition to the ground state of the neutral shifted to match the calculated VDEs. The rest of sticks at higher EBE in simulations correspond to the excited states of the neutral at the anion geometry (for simulated spectra for other isomers, see Figure S3).

Numerous conformers, in both anionic and neutral charge states, were identified by initially systematically sampling a large set of torsional angle combinations followed by geometry optimization (see Figures S1 and S2), with the four lowest ones displayed in Figure 2. Their relative energies (REs), theoretically predicted ADEs, and VDEs are listed in Table 1, along with the experimental values for comparison. The top 7 conformers—**S_1_**–**S_7_** and **S_10_**—all have predicted ADEs of 4.7–4.9 eV, which correlates well with the experimental value of 4.95 eV. Among them, only the top 4 conformers, **S_1_**–**S_4,_** possess VDE values (5.26–5.29 eV) that are closely matched to the experimental value of 5.30 eV. The first two conformers are nearly isoenergetic and should contribute most to the experimental spectrum at low temperatures, while the rest of the high-lying isomers make negligible contributions. Further evidence for this comes from their simulated spectra calculated with time-dependent CAM-B3LYP DFT calculations, which exhibit a pattern similar to the experiment (Figure 3). 

**Figure 4:**
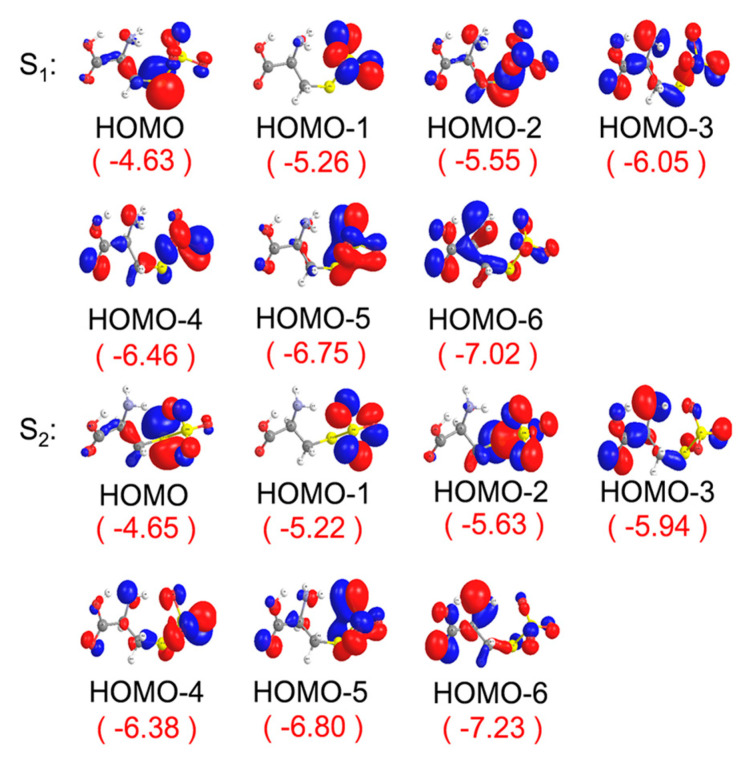
Molecular orbital (MO) analysis for conformers **S_1_** and **S_2_** of the cysteine perthiosulfonate anion [cysS-SO_3_]^−^. The MO energy levels are shown in parentheses (in eV).

**Figure 5:**
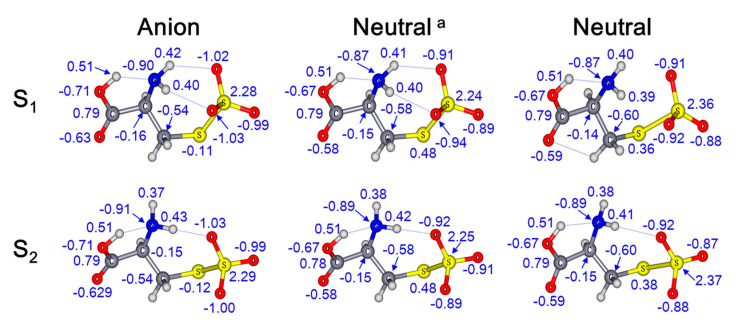
NPA charges on C, N, O, and S atoms and the H atom in hydrogen bonds (blue lines) for the most stable conformers, **S_1_** and **S_2,_** of [cysS-SO_3_]^−^ and their neutral counterparts (^a^ NPA charges for the neutrals with the anion geometry).

Figure 4 shows the highest occupied molecular orbitals for isomers **S_1_** and **S_2_**. Based on the generalized Koopman theorem [11], the first band X is associated with a detaching electron from the HOMO orbital, mainly consisting of the lone pair *π* from the linkage –S– atom. For band A, it corresponds to the electron photodetachment from HOMO-1/HOMO-2 orbitals, with the electron mainly residing on the –S–SO_3_ moiety. As for the higher energy band B, it primarily arises from the HOMO-4/HOMO-5 orbitals largely delocalized over the molecule. Natural population analysis (NPA) [12] calculations indicate that the majority of excess negative charge (~60%) is detached from the linkage –S– atom (Figure 5). HOMO and NPA charge analysis suggest that the most reactive unit of the molecule susceptible to electrophilic attacks is the linkage S atom, not the negative charge carriers (O atoms of carboxylic and sulfite groups, N of amino).

To obtain further insight into H-bond interactions, natural bond orbital (NBO) analyses were performed for conformers **S_1_** and **S_2_**. From the theoretical point of view, the H-bond effect can be viewed as the interaction between the lone pair (LP) and anti-bonding orbitals (BD*) of X−H (X = O or N), which can be quantified by the so-called noncovalent delocalization energy lowering △E^(2)^_ij_ [13] due to interactions between the donor orbital i and acceptor orbital j.

**Figure 6:**
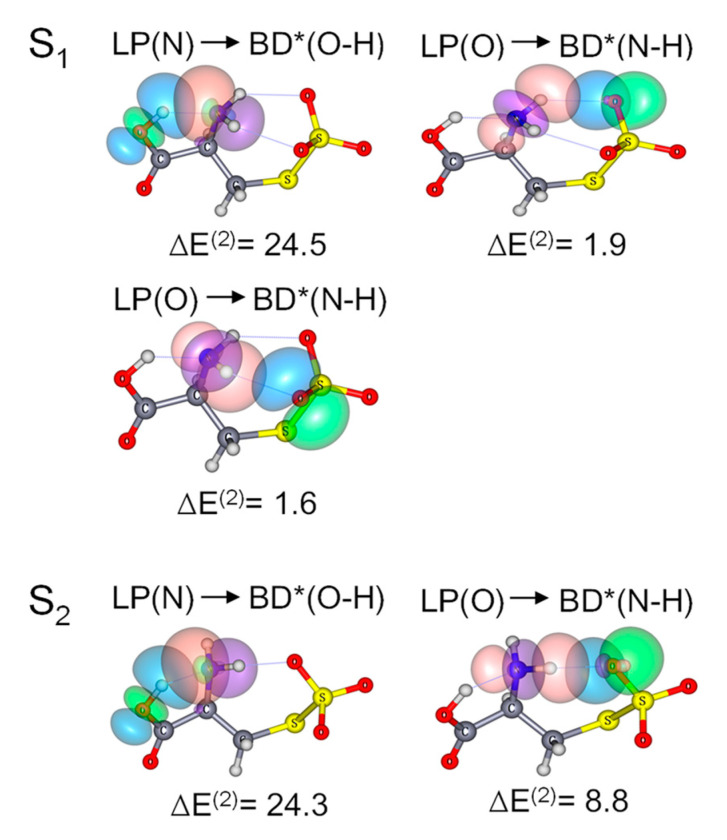
NBO analysis for conformers **S_1_** and **S_2_** of [cysS-SO_3_]^−^. (Unit: kcal/mol).

Figure 6 shows the results of such analysis for each HB along the O−H⋯⋯N−H⋯⋯O−S chain. A medium donor–acceptor interaction is observed in conformer **S_1_** with delocalization energy of △E(2) = 24.5 kcal/mol between carboxylic O−H and NH_2_, and much weaker, with a delocalization energy of △E(2)~1.6–1.9 kcal/mol between NH_2_ and SO_3_ groups. In the case of **S_2_**, the delocalization energy for the O–H⋯N H-bond is 24.3 kcal/mol, comparable with that in **S_1_**. As for the N–H⋯O H-bond, the delocalization energies are weaker again (△E^(2)^ = 8.8 kcal/mol). 

**Figure 7:**
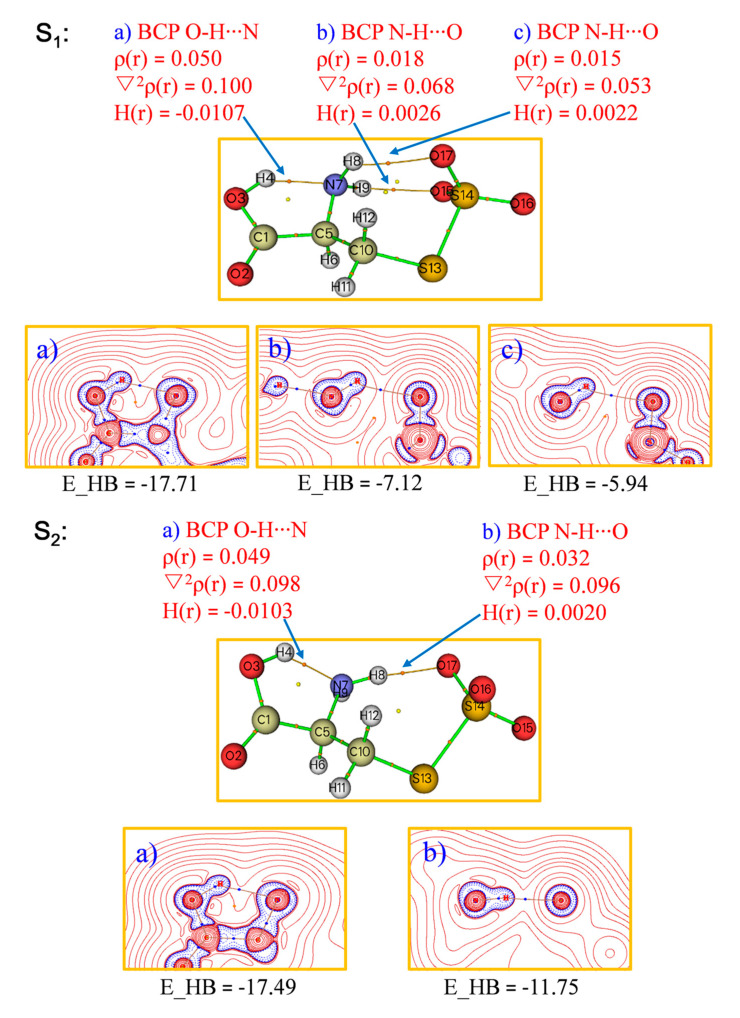
QTAIM analysis for the most stable conformers, **S_1_** and **S_2,_** of the cysteine perthiosulfonate anion [cysS-SO_3_]^−^.

Additional analysis of HB interactions in conformers **S_1_** and **S_2_** was performed with the QTAIM [14] approach, where bond critical points (BCPs) clearly reveal hydrogen bonding interactions between NH_2_ and SO_3_ moieties as well as between the COOH and NH_2_ (Figure 7 and Table S2). Based on the theory proposed by Rozas et al., the hydrogen-bonding interaction can be sorted according to density of all electrons ρ(r), electronic energy density H(r) at BCPs, and HB energy (E*_HB_) as follows [15,16]:

(1) ▽^2^ρ(r_BCP_) > 0 and H_BCP_ > 0 (E*_HB_ < 12.0 kcal/mol) suggest weak HBs and a primarily electrostatic character.

(2) ▽^2^ρ(r_BCP_) > 0 and H_BCP_ < 0 (12.0 kcal/mol < E*_HB_ < 24.0 kcal/mol) suggest medium H-bonds and a partially covalent character.

(3) ▽^2^ρ(r_BCP_) > 0 and H_BCP_ < 0 (E*_HB_ > 24.0 kcal/mol) suggest strong H-bonds and a pronounced covalent character.

According to the above classifications, isomer **S_1_** has one medium O–H⋯N HB and two weak N–H⋯O HB, while **S_2_** features one medium O–H⋯N and one weak N–H⋯O HBs, in line with the above NBO analyses. 

It is worth noting that the global minimum of the anion **S_1_** is stabilized through the formation of three intramolecular hydrogen bonds (HBs); one of them comes from amino N playing the acceptor role of HB (O−H⋯⋯N) and the rest from two amino H acting as HB donors to −SO_3_. The amino group thus serves as a multi-hydrogen-bonding relay accepting one proton from carboxylic and donating two protons to sulfite groups. The other lowest-lying isomers (**S_2_**–**S_4_**) all feature two intramolecular HBs of O−H⋯⋯N−H⋯⋯O−S-type binding motifs (Figure 2). The NPA charge analysis indicates that the excess negative charge resides mainly on the –SO_3_ moiety. For **S_1_**, this charge distribution is symmetric across the two O atoms (−1.02 e), which facilitates the formation of double N–H⋯O HBs. In comparison, the asymmetric charge distribution between two O atoms (−1.03 e and −1.00 e) in **S_2_** lead to only a single N–H⋯O HB (Figure 5).

**Figure 8:**
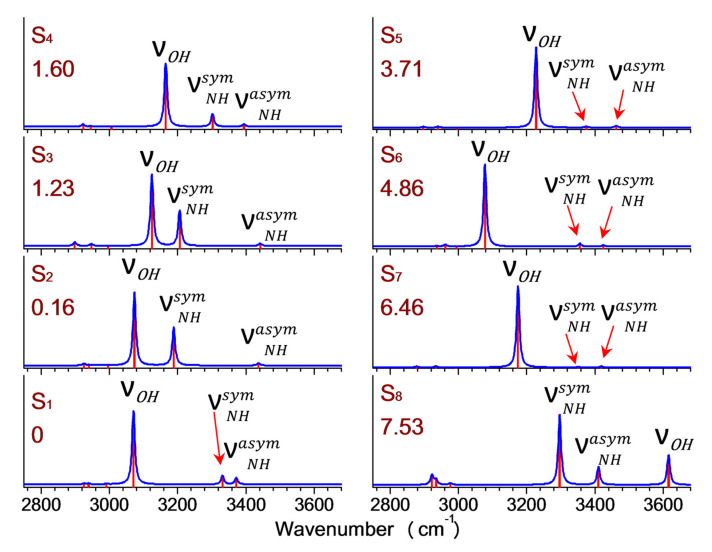
Simulated IR spectra at the level of CAM-B3LYP in the range of 2800 cm^−1^ and 3700 cm^−1^ for **S*_i_*** (*i* = 1–8) (FWHM = 10 cm^−1^, scaled by 0.954) with their relative energies (kcal/mol) noted.

To better distinguish these low-lying conformers, we calculated the infrared (IR) spectrum for each isomer to establish a fingerprint vibrational region for further IR-based experiments. The IR spectra were simulated at the CAM-B3LYP/maug-cc-pVTZ level using harmonic approximation. The full range of 0–4000 cm^−1^ IR spectra for conformers **S_1_**–**S_8_** are summarized in Figure S4 and Table S1 of Supplementary Materials. In the low-energy region (0–1800 cm^−1^), the IR spectra for **S_1_**–**S_4_** are rather similar, and this spectral region may not be ideal for isomers’ identification. Interestingly, the simulated IR spectra in the range of 2800–3700 cm^−1^ for conformers **S_1_**–**S_4_** display distinct spectral patterns ascribed to the N–H and O–H stretching modes (Figure 8). There is only one intense peak for **S_1_**, at 3071 cm^−1^, corresponding to the O–H stretching mode. For **S_2_**, two intense peaks are located at 3074 and 3189 cm^−1^, assigned to the O–H stretching mode and N–H symmetric stretching mode, respectively. For conformers of **S_3_** and **S_4_**, both have visible double peaks, which are somewhat similar to **S_2_**, but with a clear spectral blueshift. For conformers **S_5_**–**S_7_**, there is one intense peak assigned to the O–H stretching mode (3228 cm^−1^ for **S_5_**, 3078 cm^−1^ for **S_6_** and 3175 cm^−1^ for **S_7_**). For **S_8_**, one intense peak at 3297 cm^−1^ is identified as the N–H stretching mode. Close examinations of the O/N–H stretching oscillations indicate that the magnitude of frequency redshifts correlates with the HB strength, in which the respective O/N–H bond serves as the HB donor. Indeed, compared to the frequency of 3616 cm^−1^ for the nearly free O–H vibration in **S_8_**, the largest red shift of −545 cm^−1^ (3071–3616) is observed in **S_1_** that has relatively strong O−H⋯⋯N HB (r_O–H⋯N_~1.77 Å). A comparable red shift of −538 cm^−1^ (3078–3616 cm^−1^) is observed in **S_6_**, due to the formation of a similarly strong HB between carboxylic O–H with the –SO_3_ group (r_O–H⋯O_~1.67 Å) in this conformer (Figure S1). 

## 3. Materials and Methods

### 3.1. Experimental and Computational Methodologies

#### 3.1.1. Negative Ion Photoelectron Spectroscopy

The experiments were performed using a size-selective, cryogenic, negative ion photoelectron spectroscopy (NIPES) apparatus coupled with an electrospray ion source [17]. Deprotonated L-cysteine S-sulfate anions [cysS-SO_3_]^−^ were produced by electrospraying ~0.1 mM solutions of L-cysteine S-sulfate acid (cysS-SO_3_H) in a mixture solvent of methanol/water (3/1 volume ratio) tuned to slightly basic conditions (pH~8) by adding 10 mM aqueous NaOH dropwise. The anions were guided by two radio frequency quadrupole ion guides followed by a 90° bender into the cryogenic 3D Paul trap set at T = 20 K, where they were accumulated and collisionally cooled with a cold buffer gas (20% H_2_ in helium) for periods of 20–80 ms before being pulsed out into an extraction zone of a time-of-flight (TOF) mass spectrometer at 10 Hz. For each NIPES experiment, the [cysS-SO_3_]^−^ anions were mass-selected and maximally decelerated before being interacted with 157 nm (7.867 eV, an F_2_ laser) or 193 nm (6.424 eV, an ArF laser) photons in the photodetachment zone of a magnetic-bottle TOF photoelectron analyzer. The laser was operated at a 20 Hz repetition rate with the ion beam off at alternating laser shots, affording shot-to-shot background subtraction. Photoelectrons were collected at nearly 100% efficiency by the magnetic bottle and analyzed in a 5.2 m-long electron flight tube. TOF spectra were recorded and converted to kinetic energy spectra calibrated by the known spectra of I^−^ [18] and Cu(CN)_2_^−^ [19]. The electron binding energy (EBE) spectra were obtained by subtracting the kinetic energy spectra from the respective detachment photon energy. The electron energy resolution is about 2%, i.e., 20 meV full width at half maximum for electrons with 1 eV kinetic energy.

#### 3.1.2. Computational Details

**Scheme 1:**
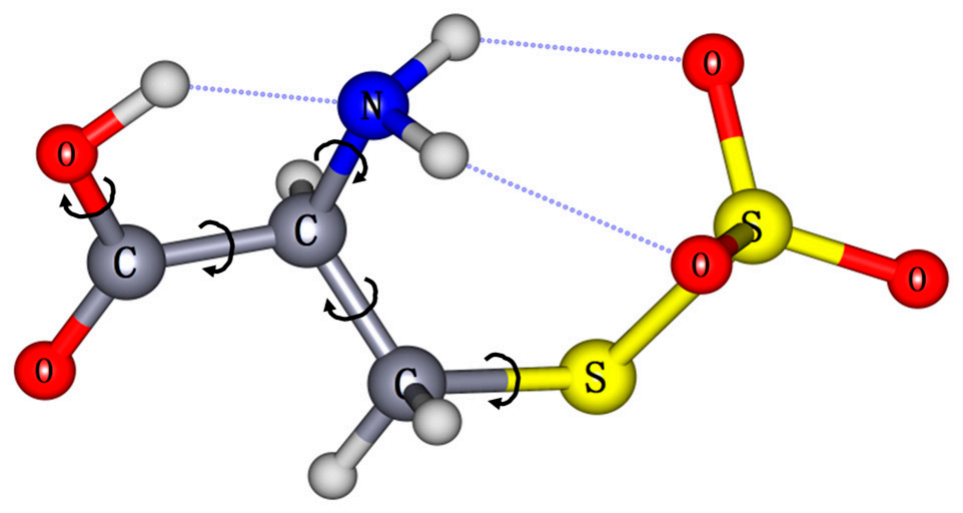
The sketch of the cysteine perthiosulfonate anion [cysS-SO_3_]^−^ and its five torsional degrees of freedom.

Due to its five torsional degrees of freedom (Scheme 1), [cysS-SO_3_]^−^ is expected to have a large manifold of plausible low-lying isomers, in which intramolecular hydrogen bonds among carboxylic, amino, and thiosulfate groups are formed whenever possible to achieve a maximum stabilization. Hence, initial conformers were systematically sampled at discrete combinational sets of torsional angles followed by geometry optimization at the B3LYP [20,21]/cc-pVTZ [22] level of theory using the Gaussian09 suite of programs [23]. The top 20 low-lying energy conformers were selected for further analysis using hybrid CAM-B3LYP [24] exchange-correction functional with the maug-cc-pVTZ basis set [22], obtained from the EMSL basis set exchange [25]. Subsequent vibrational frequency analysis was conducted at the same level to confirm that the true minima were found and to compute zero-point energies (ZPEs) with resulting frequencies scaled by a factor of 0.954 [26]. Adiabatic detachment energies (ADE) were calculated as the energy difference between the neutral radical and corresponding anion at their respective optimized structures, including the ZPE corrections. Vertical detachment energies (VDE) were calculated as the energy difference between the neutral and anion, both at the anion geometry [27].

## 4. Conclusions

We have characterized various conformers and H-bonded patterns of L-cysteine S-sulfate anion [cysS-SO_3_]^−^ in detail for the first time via a joint ion spectroscopic and theoretical study. An intramolecular HB chain, –COO–H⋯NH_2_⋯SO_3_^−^, has been revealed to exist in all low-lying energy isomers. For the most stable conformer, **S_1_**, an intramolecular HB network is revealed, where the –NH_2_ group acts as a double donor to form two HB with the sulfite group and behaves as an HB acceptor to form a strong O–H⋯N HB with the carboxylic group. The characteristics of the HB network are further quantified by NBO and QTAIM theory, suggesting that O–H⋯N HB can be classified as a medium-strong one compared to the weak N–H⋯O HBs. The observed hydrogen-bonding network and binding motifs could be of general importance for understanding molecular mechanisms of protein post-translational modifications. 

## Figures and Tables

**Table 1:** Comparison of experimental VDE and ADE values to CAM-B3LYP calculated ones for the top 20 lowest-energy isomers of the cysteine perthiosulfonate anion [cysS-SO_3_]^−^.

Conformers.	Relative Energy [kcal/mol]	ADE [eV]		VDE [eV]	
		Theo. ^a^	Exp. ^b^	Theo. ^a^	Exp. ^b^
**S_1_**	0.00	4.87	4.95	5.26	5.30
**S_2_**	0.16	4.88		5.27	
**S_3_**	1.23	4.80		5.26	
**S_4_**	1.60	4.82		5.29	
**S_5_**	3.71	4.80		5.55	
**S_6_**	4.86	4.70		5.56	
**S_7_**	6.46	4.90		5.55	
**S_8_**	7.53	4.63		4.97	
**S_9_**	7.79	4.64		4.99	
**S_10_**	8.12	4.83		5.54	
**S_11_**	8.28	4.65		5.04	
**S_12_**	8.33	4.62		5.00	
**S_13_**	8.37	4.64		5.03	
**S_14_**	8.53	4.37		5.01	
**S_15_**	8.82	4.41		5.05	
**S_16_**	9.04	4.65		5.06	
**S_17_**	9.22	4.59		4.97	
**S_18_**	9.26	4.59		4.94	
**S_19_**	9.57	4.60		4.99	
**S_20_**	9.61	4.51		4.95	

^a^ Calculated at the CAM-B3LYP/maug-cc-pVTZ level including zero point energy (ZPE) corrections for ADEs; ^b^ experimental uncertainty is 0.1 eV.

## Data Availability

The data presented in this study are available on request from the corresponding author.

## Supplementary Materials

The following supporting information can be downloaded at: https://www.mdpi.com/article/10.3390/ijms24021682/s1.
