# Supplementary material for: Properties of Gaseous Deprotonated L-Cysteine S-Sulfate Anion [cysS-SO3]−: Intramolecular H-Bond Network, Electron Affinity, Chemically Active Site, and Vibrational Fingerprints
Source: Int J Mol Sci. 2023 Jan 14;24(2):1682. doi: 10.3390/ijms24021682 (PMC9862062; doi:10.3390/ijms24021682)
Supplement: Supplementary file 1 [file ijms-24-01682-s001.zip › ijms-2107242-supplementary.pdf]

Supplementary Materials for

Properties of Gaseous Deprotonated L-Cysteine S-Sulfate Anion $[cysS\text{-}SO_3]^-$: Intramolecular H-Bond Network, Electron Affinity, Chemically Active Site, and Vibrational Fingerprints

Qiaolin Wang,[a,†] Zhengbo Qin,[a,†] Gaolei Hou,[b] Zheng Yang,[b] Marat Valiev,[c,]* Xue-Bin Wang,[b,]* Xianfeng Zheng,[a] Zhifeng Cui[a]

[a.] *Anhui Province Key Laboratory of Optoelectric Materials Science and Technology, Anhui Normal University, Wuhu, 241000, China.*

[b.] *Physical Sciences Division, Pacific Northwest National Laboratory, 902 Battelle Boulevard, P. O. Box 999, MS K8-88, Richland, Washington 99352, USA.*

[c.] *Environmental Molecular Sciences Laboratory, Pacific Northwest National Laboratory, P. O. Box 999, Richland, WA 99352, USA.*

* Authors to whom correspondence should be addressed.

Electronic mails: marat.valiev@pnnl.gov (M. Valiev),

xuebin.wang@pnnl.gov (X.-B. Wang)

[†]Qiaolin Wang and Zhengbo Qin contributed equally to this work.

Table S1. Calculated vibrational frequencies (cm^{-1}) for the top eight low-lying isomers $\mathbf{S}_i$ (i=1-8) at the CAM-B3LYP/maug-cc-pVTZ level.

Mode	S_1	S_2	S_3	S_4	S_5	S_6	S_7	S_8
Q_1	35	30	29	42	22	29	29	25
Q_2	58	47	49	60	51	88	31	40
Q_3	109	77	74	71	71	117	67	61
Q_4	117	134	109	129	96	148	85	107
Q_5	148	146	145	139	177	167	157	135
Q_6	192	193	191	173	195	202	181	193
Q_7	219	209	263	213	211	231	196	201
Q_8	276	281	283	280	263	257	239	266
Q_9	307	308	292	300	276	278	278	279
Q_{10}	334	329	320	347	304	282	284	305
Q_{11}	355	362	358	365	327	304	320	331
Q_{12}	373	371	382	383	347	349	360	351
Q_{13}	427	417	483	417	373	381	369	391
Q_{14}	496	496	497	495	495	505	495	490
Q_{15}	509	507	506	505	499	506	498	496
Q_{16}	516	524	518	510	511	581	506	508
Q_{17}	586	596	570	591	578	593	574	556
Q_{18}	596	627	597	597	591	616	578	590
Q_{19}	682	671	670	685	697	678	708	627
Q_{20}	762	768	741	759	737	757	763	676
Q_{21}	818	812	796	794	762	797	777	773
Q_{22}	864	868	874	884	779	864	783	809
Q_{23}	931	942	910	909	821	879	877	855
Q_{24}	968	961	949	929	901	897	953	915
Q_{25}	983	973	984	986	991	969	972	985
Q_{26}	1064	987	1011	1041	1012	987	994	997
Q_{27}	1100	1091	1070	1095	1076	1070	1098	1083
Q_{28}	1108	1113	1142	1115	1141	1141	1127	1103
Q_{29}	1168	1170	1168	1167	1177	1155	1186	1157
Q_{30}	1187	1184	1178	1193	1196	1175	1204	1183
Q_{31}	1199	1209	1203	1199	1204	1231	1214	1202
Q_{32}	1219	1228	1222	1228	1221	1242	1230	1218
Q_{33}	1254	1265	1263	1244	1237	1269	1266	1238
Q_{34}	1280	1280	1275	1286	1323	1293	1306	1270
Q_{35}	1350	1333	1342	1354	1339	1352	1333	1295
Q_{36}	1401	1410	1399	1402	1383	1375	1368	1387
Q_{37}	1420	1415	1413	1406	1414	1408	1429	1401
Q_{38}	1618	1626	1607	1605	1553	1606	1598	1594
Q_{39}	1760	1760	1763	1765	1741	1740	1735	1755
Q_{40}	2926	2926	2899	2923	2897	2936	2878	2923
Q_{41}	2939	2941	2948	2945	2941	2962	2934	2935
Q_{42}	2991	2997	2997	3007	2997	2995	2993	2977
Q_{43}	3071	3074	3124	3165	3228	3078	3175	3297
Q_{44}	3332	3188	3206	3303	3375	3356	3351	3410
Q_{45}	3372	3437	3442	3394	3463	3425	3419	3616

Table S2. QTAIM parameters for the top two isomers calculated at the CAM-B3LYP/maug-cc-pVTZ level.

		Hydrogen-bond type	BCP ρ(r)	BCP ∇²ρ(r)	BCP H(r)	E_HB
			[a.u.]	[a.u.]	[a.u.]	[kcal/mol]
**S_1**	a	O-H···N	0.050	0.100	-0.0107	-17.71
	b	N-H···O	0.018	0.068	0.0026	-7.12
	c	N-H···O	0.015	0.053	0.0022	-5.94
**S_2**	a	O-H···N	0.049	0.098	-0.0103	-17.49
	b	N-H···O	0.032	0.096	0.0020	-11.75

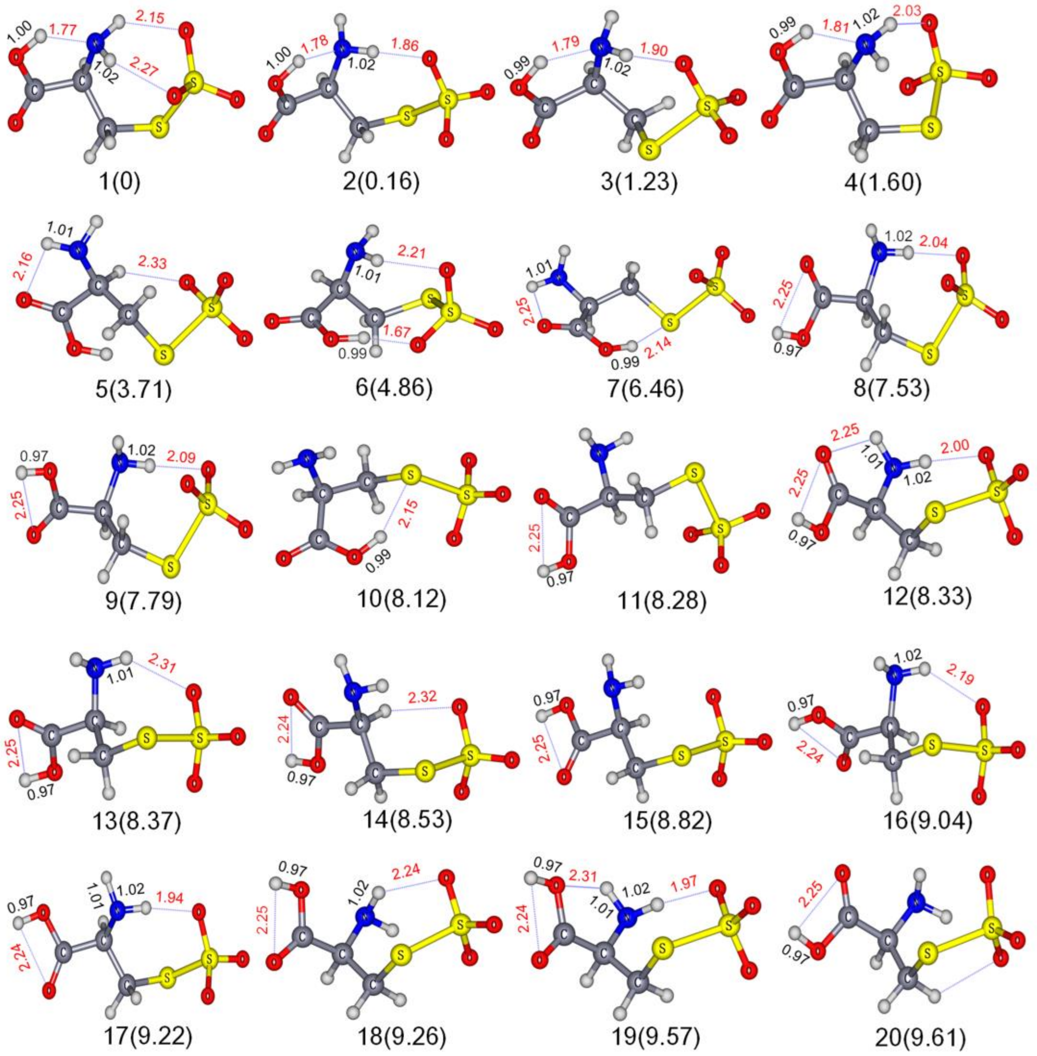

Figure S1. Top twenty conformers of cysteine perthiosulfonate anion $[cysS\text{-}SO_3]^-$ calculated at the CAM-B3LYP/maug-cc-pVTZ level. The relative energies at 0 K (kcal/mol, including ZPE) are indicated in parenthesis below each structure.

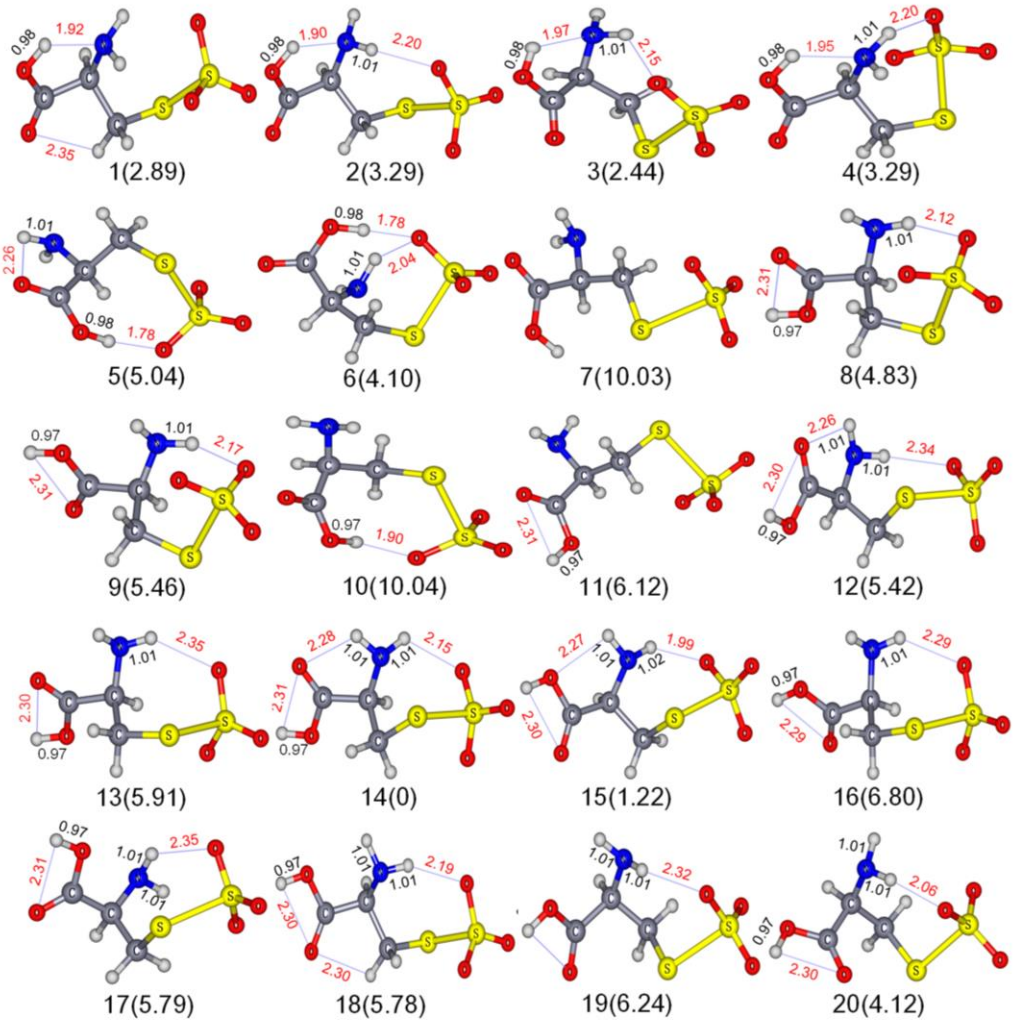

Figure S2. Top twenty conformers of $[cysS\text{-}SO_3]^{\bullet}$ radical calculated at the CAM-B3LYP/maug-cc-pVTZ level. The relative energies at 0 K (kcal/mol, including ZPE) are indicated in parenthesis below each structure.

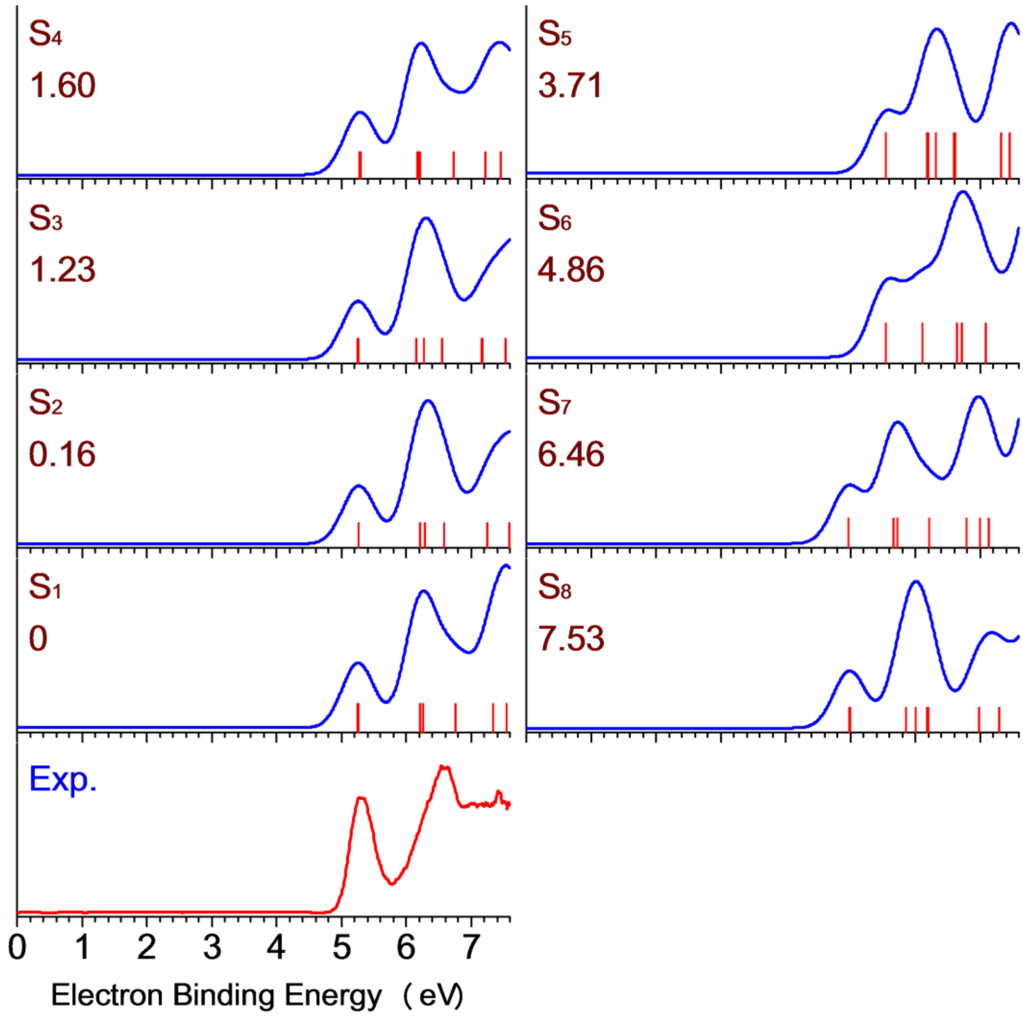

Figure S3. Simulated photoelectron spectra at the level of TD-CAM-B3LYP for $\mathbf{S}_i$ (i = 1-8) (FWHM = 0.35 eV) along with the experimental one for comparison.

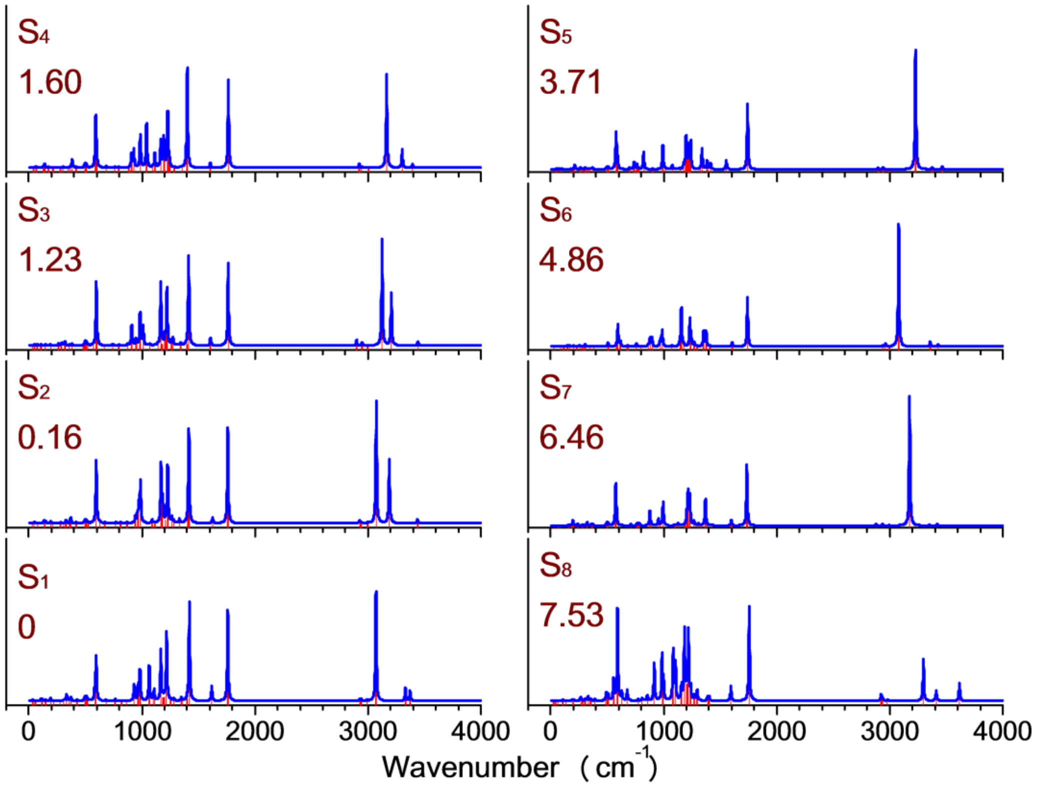

Figure S4. Simulated IR spectra at the level of CAM-B3LYP in the range of 0 cm^{-1} and 4000 cm^{-1} for $\mathbf{S}_i$ (i = 1-8) (FWHM = 10 cm^{-1}, scaled by 0.954).
